# Gyrator Based on Magneto-elastic Coupling at a Ferromagnetic/Piezoelectric Interface

**DOI:** 10.1038/s41598-017-00960-9

**Published:** 2017-04-12

**Authors:** Swapnil Bhuktare, Arnab Bose, Hanuman Singh, Ashwin A. Tulapurkar

**Affiliations:** Department of Electrical Engineering, Indian Institute of Technology-Bombay, Powai, Mumbai 400076 India

## Abstract

A gyrator is a non-reciprocal two port device with 180° phase shift in the transmissions between two ports. Though electromagnetic realizations of gyrators have been well studied, devices based on other forms of interaction are relatively unexplored. Here we demonstrate a device in which signal is transmitted via magneto-elastic coupling, can function as a gyrator. The device is built on a piezoelectric substrate: one port of this device has interdigital transducers (IDTs) and the other port has a periodic array of nickel/gold lines. When the magnetizations of Ni lines are excited into precession by magnetic field generated by passing oscillating current through the gold lines, they emit phonons in the form of surface acoustic waves (SAW) due to the magneto-elastic coupling between Ni and substrate. The emitted SAW can be detected at the other end by the IDTs. Conversely, when SAW is incident on Ni lines from IDTs, the magnetization undergoes precession and can be inductively detected by Au lines. The broken time reversal symmetry of the system due to the presence of ferromagnet gives rise to the non-reciprocal transmission between the two ports. These devices could function as novel building blocks for phonon based information processing.

## Introduction

Spin mechanics deals with the coupling between the spin and the mechanical degrees of freedom. The macroscopic-scale Barnett or Einstein–de Haas effects originating from this were discovered long back^1, 2^. These magnetoelastic interactions between magnons and phonons were studied theoretically first by Akhiezer and Kittel and then verified experimentally by several groups^3–5^. The manifestation of these mechanical effects in synthetic multiferroic and other systems at nanoscales and their possible technological applications ranging from energy conversion devices to memory or logic operations have generated renewed interest in the scientific community^6–14^. The phenomenon of magnetostriction or its Onsager equivalent, the inverse magnetostriction can be used to control the magnetization by application of strain. Synthetic multiferroic systems comprising magnetostrictive material deposited on piezoelectric substrate are ideal candidates for spin-mechanics effects. The surface acoustic waves (SAW) generated in piezoelectric substrate can be used to manipulate the magnetization of magentostrictive material.

Surface acoustic waves (SAW) can be efficiently generated in piezoelectric substrate with the help of interdigital transducer (IDT) structures. A typical SAW device comprises two sets of IDTs. The input IDT converts the periodic voltage signals into SAW which travels along the surface. The output IDT converts the waves into electrical signals again and is used for detection^15^. The interaction between SAW and a ferromagnet has been studied recently by measuring the transmission between the two IDTs with a FM deposited between them^16–19^. Ferromagnetic resonance (FMR) excited by surface acoustic waves was demonstrated experimentally in Ni thin films by Weiler *et al*.^16, 17^ and in GaMnAs dilute ferromagnetic semiconductor films by Thevenard *et al*.^18^ recently. Magnetization switching with either pure SAW or with spin-transfer torque assisted by SAW was studied theoretically by Thevenard *et al*.^20^ and Biswas *et al*.^21^ respectively. Ultrafast magnetization switching with SAW was demonstrated experimentally by Davis *et al*.^22^ and its use for nanomagnetic logic operation was explored by Sampath *et al*.^23^. This new approach of magnetization switching with SAW is also efficient in terms of power dissipation and could soon find potential technological applications.

## Results

**Figure 1:**
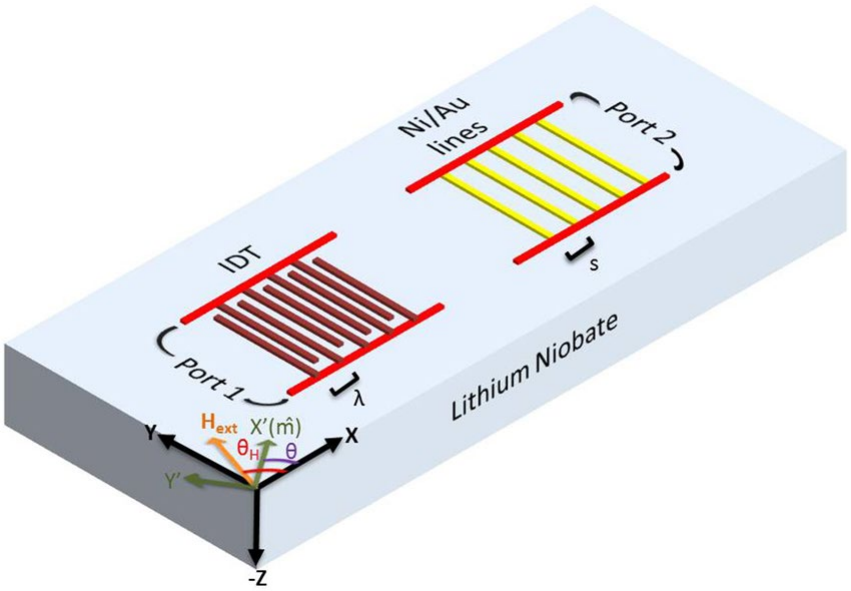
The schematic of the device. Ni/Au lines are on one side and IDT is on another side. The periodicity of the Ni/Au lines s was either λ or 0.5 λ. The Ni lines undergo FMR and generate SAW which can be detected by the IDT at the other side.

In this work, we demonstrate the inverse effect i.e. generation SAW by oscillating magnetization. All the experiments were done at room temperature on 128° Y cut lithium niobate substrates with a wave velocity of 3980 m/s. The device fabrication was done with standard lithography (e beam and optical), deposition and lift off techniques. To ensure that the Ni films deposited are indeed interacting with the surface waves, we first performed the acoustically driven ferromagnetic resonance (ADFMR) experiment, the results of which are shown in the supplementary material. For the inverse experiment i.e. generation of SAW by FMR, we fabricated IDT on one side and an array of 100 lines of Ni (20 nm)/Au (80 nm) on the other side, as shown schematically in Fig. 1. Each Ni/Au line is about 400 nm wide and 90 um long. The IDT design is same as used for the ADFMR experiment (λ~2 μm). Two sets of devices were fabricated; one in which the periodicity of Ni/Au lines was same as that of the IDT (s = λ) and the other in which it was half of the IDT(s = 0.5 λ).

### FMR study

**Figure 2:**
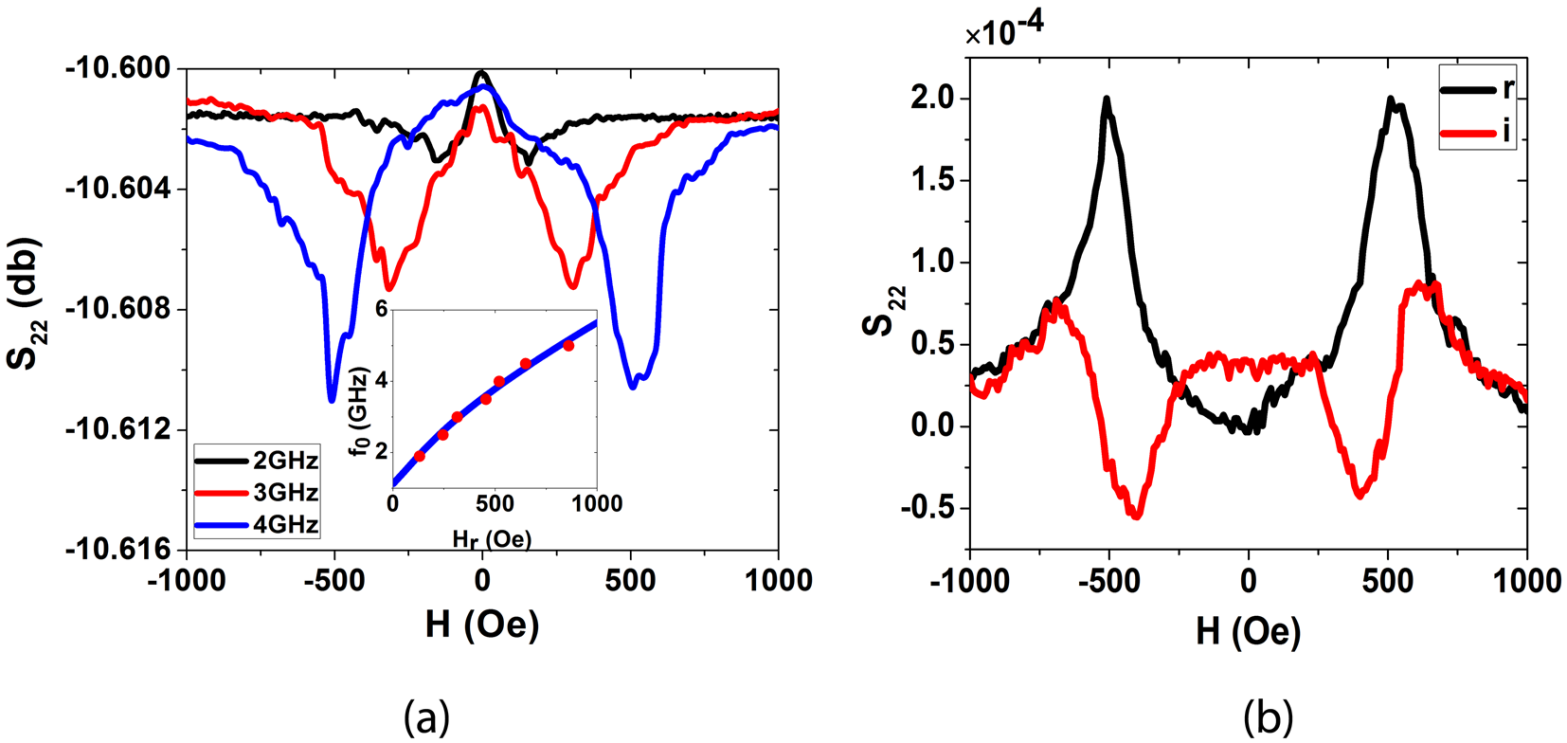
FMR study: (**a**) The FMR signal of the device for different frequencies, the magnetic field was applied at an angle of 45° for this measurement. The inset shows the Kittel’s plot wherein the red points show the experimental data of resonance frequency as a function of magnetic field and the blue curve is obtained from Kittel’s relation. (**b**) The real and the imaginary parts of the S_22_ signal for 4 GHz frequency, the black curve marked as ‘r’ and the red curve marked as ‘i’ show the real and imaginary parts respectively.

As a first step, we measured the FMR of Ni/Au lines. This corresponds to the measurement of S_22_ scattering parameter by using a vector network analyzer (VNA). When rf voltage is applied to port 2, most of the rf current flows in the top Au part due to its higher thickness and conductivity than Ni. The resultant rf Oersted magnetic field along x direction, drives the FMR of Ni. The inductive voltage from the oscillating magnetization is picked up by the Au lines. The external magnetic field was applied at an angle of 45°. The S_22_ signals measured with the VNA for different frequencies are shown in Fig. 2(a). The symmetric dips correspond to the FMR. The resonance frequency as a function of external magnetic field is shown in the inset of Fig. 2(a). The resonance frequency follows Kittel’s relation:

1 $${f}_{0}\approx (\gamma /2\pi )\sqrt{(H{^{\prime} }_{ext}+H{^{\prime} }_{//})(H{^{\prime} }_{ext}+H{^{\prime} }_{//}+H{^{\prime} }_{\perp })}$$

where, ${H^{\prime} }_{ext}={H}_{ext}\,\cos \,(\theta -{\theta }_{H}),{H^{\prime} }_{//}={H}_{//}({\cos }^{2}\,\theta -{\sin }^{2}\,\theta ),{H^{\prime} }_{\perp }={H}_{\perp }+{H}_{//}{\sin }^{2}\,\theta$ where *H*_//_ and *H*_*⊥*_ denote in-plane and out-of-plane anisotropy fields, θ_H_ denotes the angle between magnetic field and x –axis, θ denotes the angle between magnetization and x-axis. The blue line in the inset of Fig. 2(a) was obtained using equation (1), with *H*_//_ = 55 Oe, *H*_*⊥*_ = 3.8 kOe and *γ* = 2.05 × 10^5^ m/(A s).

### Generation of SAW by magnetization precession and gyrator behavior

**Figure 3:**
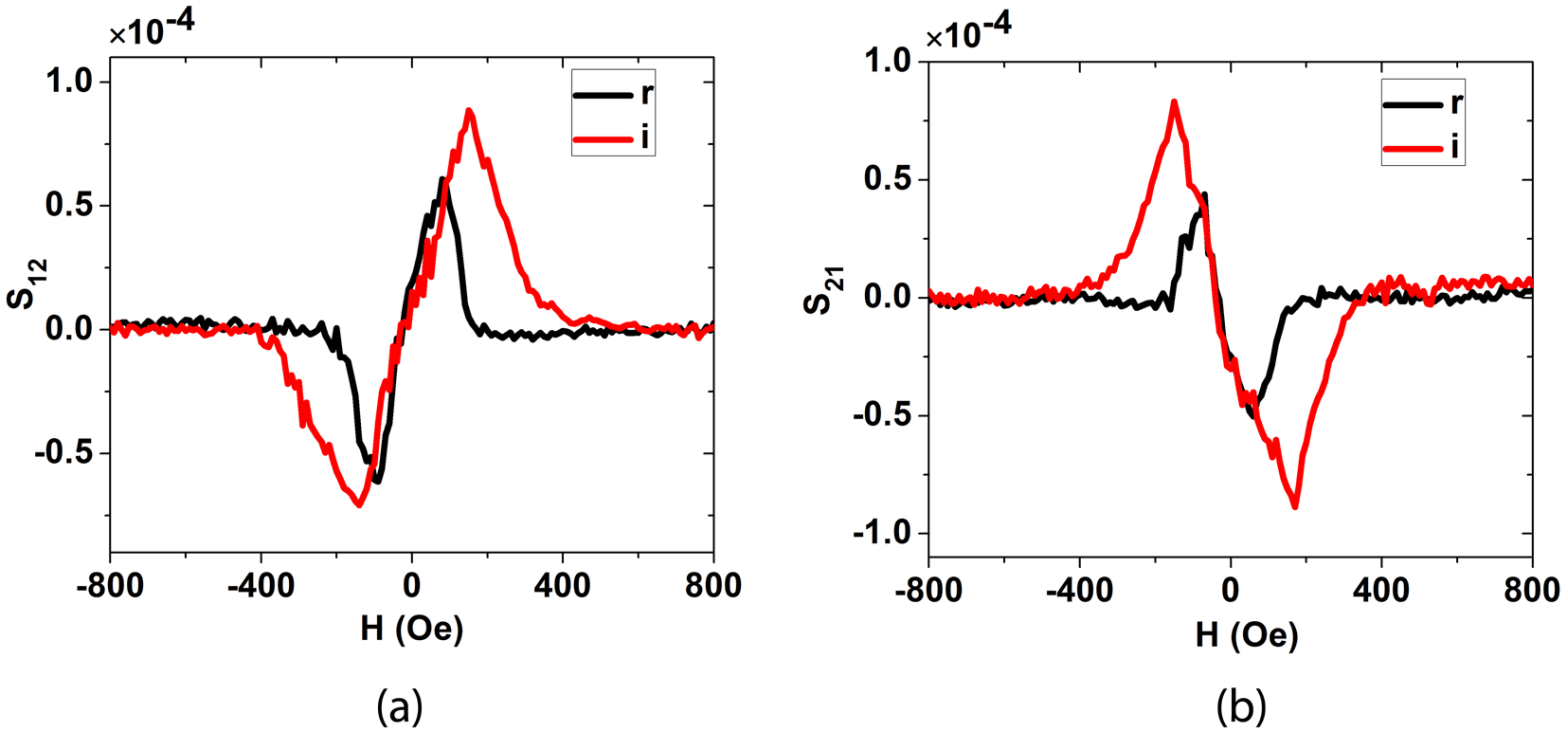
SAW generation: the field was applied at an angle of 45° for all these measurements. (**a**) Real and imaginary parts of S_12_. (**b**) Real and imaginary parts of S_21_.

We now present results on two port S-parameter measurements with VNA on device with s = λ. We fix the frequency (1.89 GHz) which matches the frequency of the IDT (which can be determined experimentally by the dip in S_11_ in this case as can be seen from Fig. S1(c)) and again sweep the magnetic field at an angle of 45°. The real and imaginary parts of S_12_ signal obtained after time gating and background subtraction are shown in Fig. 3(a). We see peaks at a certain positive field value but dips at negative field value for both of them. (The magnitude of S_12_ signal obtained from Fig. 3(a) is shown in Fig. S4). We can see two clear peaks in S_12_ at the positions of resonance fields. This indicates that signal from port 2 gets transmitted to port 1 when the ferromagnetic material is undergoing FMR. We ascribe this to the generation of SAW from each Ni line due to magneto-elastic coupling. The waves generated by each line interfere constructively as the separation between the lines is λ. The real and imaginary parts for S_21_ are shown in Fig. 3(b). The peak and the dip positions are reversed, which implies that the magnitude of S_12_ remains the same but there is a phase change of 180° or in other words the device shows non-reciprocal behavior. (The generalized time reversal relation: S_12_(H) = S_21_(−H) is however obeyed as can be seen from Fig. 3(a) and (b).) The S_21_ signal arises due the following reason: The voltage applied to IDTs (port 1) generate SAW, which creates an effective rf magnetic field on Ni lines^11, 12^. At certain value of the external field, Ni magnetization undergoes FMR. The phase of the rf magnetic field is the same at all Ni lines as they are separated by a distance of λ. Thus magnetization of all Ni lines oscillate in phase and the induced current in all Au lines add constructively, which is detected as S_21_ signal.

The S_12_ signal for some other frequency (3 GHz) is shown in Fig. S6(a) in supplementary material. The FMR field for 3 GHz is ± 315 Oe (Fig. 2(a)). We don’t see any clear peak or dip in S_12_ around this field value. This is because even if we have FMR and generation of SAW at 3 GHz from each Ni line, the corresponding wavelength (λ = 1.33 μm) does not match the periodicity of transmitting and receiving ports.

**Figure 4:**
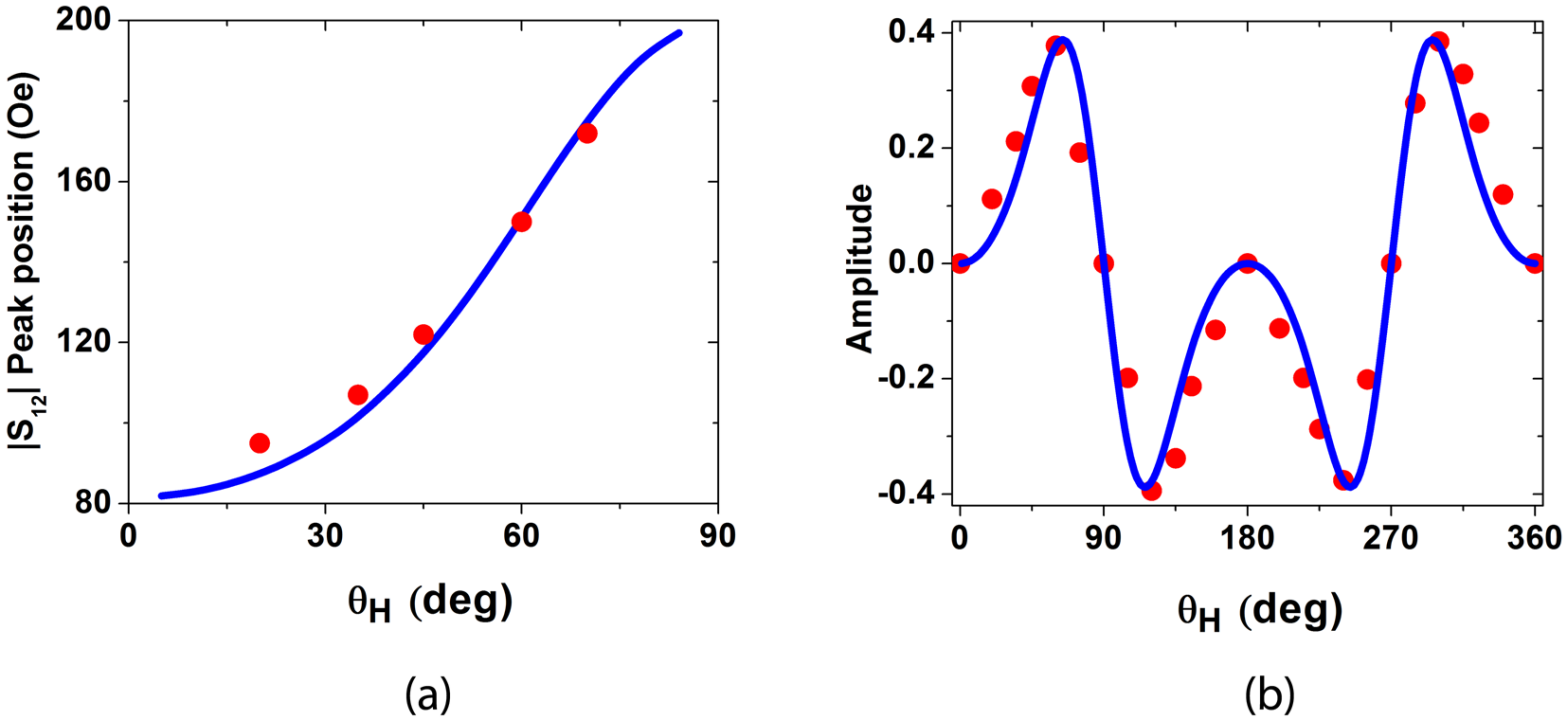
Study of variation of applied magnetic field direction: (**a**) Position of |S_12_| peak vs. angle of applied magnetic field. The red points show the experimental data and the continuous blue curve is obtained from eqn. S16. (**b**) Variation of the amplitude of |S_12_|, the red points show the scaled experimental data (for the sake of clarity) and the continuous blue curve shows the sin^2^ (θ)cos (θ) curve.

We then carried out above measurements (f = 1.89 GHz) with magnetic field swept at different angles. We couldn’t see any signal for θ = 0° or θ = 90°. The peak position of |S_12_| with respect to angle is shown in Fig. 4(a). The position closely follows the position of the FMR which again confirms that the signals are arising solely due to the FMR. The amplitude of the peak follows a sin^*2*^(*θ*)cos(*θ*) dependence which is shown in Fig. 4(b). (see supplementary material for explanation). This four-fold symmetric behavior is similar to the one reported in acoustically driven ferromagnetic resonance experiments^16, 17^. Another interesting point is that the magnetic field as plotted in Fig. 4(a) increases with angle, which shows that x-axis is the easy axis of Ni lines, though the shape anisotropy would have preferred y-axis. (This might happen because the film is grown on a single crystal substrate). We verified this from S_22_ signal as well. We measured S_22_ at 4 GHz and found that the position of peak in the |S_22_| shifted to higher magnetic fields with increasing θ_H_. The blue curve in Fig. 4(a) is obtained by numerically calculating the magnetic field at which S_12_ is maximum (eqn. S16), using the same values of *H*_//_ and *H*_*⊥*_ as used in Fig. 2(a).

We then measured S-parameters of the device with s = λ/2. The results are shown in Fig. S6(b) in supplementary material. Even if we have FMR and generation of surface acoustic waves from each Ni line, because of the periodicity, there is destructive interference and no wave propagates towards the IDTs (i.e. S_12_~0). Conversely, when a SAW is incident from the IDTs on the array of Ni/Au lines, the magnetization of adjacent Ni lines oscillate 180° out of phase, and no net current is induced in the Au lines (i.e. S_21_~0).

## Discussion

The non-reciprocal behavior of our device can be understood if we compare the ADFMR to the Oersted magnetic field driven FMR (which corresponds to S_22_ measurement). The real and imaginary S_22_ parameters as a function of dc magnetic field are shown in Fig. 2(b). One sees the expected peak in the real part and dispersion of the imaginary part. We can see from the figure that S_22_(H) = S_22_(−H). All this can be well explained by LLG equation. (See eqn. S3 in the supplementary information. Applying −H corresponds to applying magnetic field H, at θ + π. Since sin^2^ (θ) = sin^2^ (θ + π), the oscillation of x-component of magnetization is unchanged, and so is the induced voltage in the Au line.) When we reverse the dc magnetic field, the equilibrium magnetization reverses but the rf magnetic field remains the same. Now consider ADFMR. Here when we reverse the dc magnetic field, even the rf magnetic field changes sign, as its generation is related to the equilibrium magnetization direction (see eqn. S2 in the supplementary information). Thus the induced voltage in case of ADFMR changes sign when dc magnetic field is reversed. Thus we expect, S_21_(H) = −S_21_(−H). Now from generalized time reversal symmetry, we should have S_12_(H) = S_21_(−H). Combining these two relations, we get S_12_(H) = −S_21_(H).

The above non-reciprocal results can also be obtained explicitly by considering how signal propagates between the two ports. Signal propagation from port 1 to 2, involves ADFMR and inductive pick up at port 2. Signal propagation from port 2 to 1, involves generation of acoustic waves by oscillating magnetization as shown schematically in Fig. S2 in the supplementary material, and then detection by IDTs.

When the magnetization of a Ni line oscillates, it generates SAW propagating along ±x direction with amplitude ε_0_ given by the following relation: (The line is assumed to be very thin (*dl* << λ) along x direction).

2 $$\varepsilon =A{b}_{1}\,{m}_{x0}\frac{d{m}_{x}}{dt}\,where\,A={z}_{0}\frac{{k}^{2}{|{c}_{x}|}^{2}\lambda }{W}{\mu }_{0}{M}_{s}vol$$

where b_1_ is the magneto-elastic coupling coefficient, m_x0_ denotes equilibrium magnetization along x axis, z_0_ is the characteristic impedance of piezoelectric substrate (LiNbO_3_ here), c_x_ is the ratio of surface voltage to displacement associated with SAW^10^, λ denotes wavelength, W denotes the length of Ni line (same as SAW beam width), M_s_ denotes saturation magnetization, and vol is the volume of the Ni line. (See supplementary information for derivation). It can be seen from the above equation, that the generation of acoustic waves depends on m_x0_. When the magnetic field is reversed, dm_x_/dt remains the same (as discussed above), but as m_x0_ changes sign, the amplitude of surface waves emitted changes sign. The voltage detected by IDTs (which corresponds to S_12_ measurement) is therefore opposite, i.e. S_12_(H) = −S_12_(−H).

In summary, we have demonstrated the generation of surface acoustic waves via oscillating magnetization. Our device shows non-reciprocal behavior which can be exploited to design acoustic gyrators. Furthermore, the FMR can as well be generated by spin currents instead of oscillating magnetic field. This can be achieved by spin Hall effect instead of the oersted field as has been done here. It will be interesting to repeat this experiment with Ni/HM lines instead of Ni/Au lines where HM is a heavy metal like Pt or Ta with significant spin orbit coupling. This could provide new pathways for interconversion of spin currents into phonons and find potential applications in the fields of acoustics^24, 25^ as well as logic devices which exploit phonons for information processing applications.

## Methods

The devices were fabricated on 128° Y cut lithium niobate substrates. The IDTs were patterned with e beam lithography using a conducting polymer to avoid charging effects in Raith 152 tool followed by thermal evaporation of Cr/Au (10 nm/50 nm) and lift off. Lithography for Ni/Au (20 nm/80 nm) lines was then done using similar recipe, and deposition was done by sputtering. The big contact pads were then patterned with optical lithography followed by thermal evaporation of Cr/Au (10 nm/100 nm) and lift off. The measurements were carried out using Agilent N5244A network analyzer. Ferromagnetic resonance measurements were done on Port 2 using S_22_ to confirm the RF excitation of Ni lines. The frequency of the operation was determined by the dip in the S_11_. Magnetic field sweep at different angles was done and the frequency spectrum was recorded. Time gating (to eliminate electromagnetic wave coupling) and background subtraction was done to get the signals. All measurements were carried out at room temperature.

## Electronic supplementary material

Gyrator Based on Magneto-elastic Coupling at a Ferromagnetic/Piezoelectric Interface
