# Supplementary material for: Gyrator Based on Magneto-elastic Coupling at a Ferromagnetic/Piezoelectric Interface
Source: Sci Rep. 2017 Apr 12;7:840. doi: 10.1038/s41598-017-00960-9 (PMC5429798; doi:10.1038/s41598-017-00960-9)
Supplement: Supplementary file 1 — Gyrator Based on Magneto-elastic Coupling at a Ferromagnetic/Piezoelectric Interface [file 41598_2017_960_MOESM1_ESM.pdf]

Supplementary Material

Gyrator Based on Magneto-elastic Coupling at a Ferromagnetic/Piezoelectric Interface

Swapnil Bhuktare,[1] Arnab Bose,[1] Hanuman Singh,[1] and Ashwin. A. Tulapurkar[1,*]

[1]*Department of Electrical Engineering, Indian Institute of Technology-Bombay, Powai, Mumbai 400076, India*

1) Acoustically driven ferromagnetic resonance

2) Generation of SAW by FMR

3) Calculation of S parameters:

4) Numerical estimation of S_{21} signal

5) About S_{12} and S_{21} spectrum shape

6) **S-parameters of the device for some other frequency and for device with s=λ/2**

1) Acoustically driven ferromagnetic resonance

The device fabrication was done with standard lithography (e beam and optical), deposition and lift off techniques. The IDT lines were patterned with e beam lithography using a conducting polymer followed by thermal evaporation of Cr/Au (10 nm/50 nm) and lift off. Ni (100 nm) big pads and Cr/Au (20 nm/100 nm) contact pads were then made in next steps using photolithography, deposition and lift off. The S-parameter measurements were carried out with vector network analyzer. The acoustically driven FMR measurements were carried out at the fundamental frequency (1.89 GHz) of SAW. The device schematic and the measurement results are shown in Fig.1.

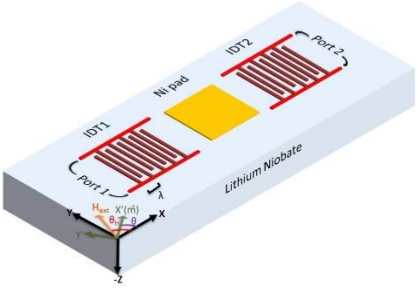

(a)

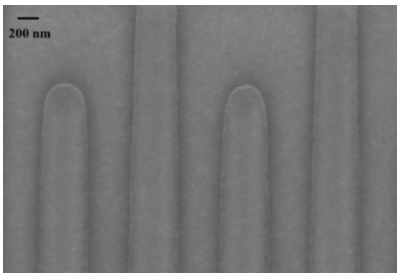

(b)

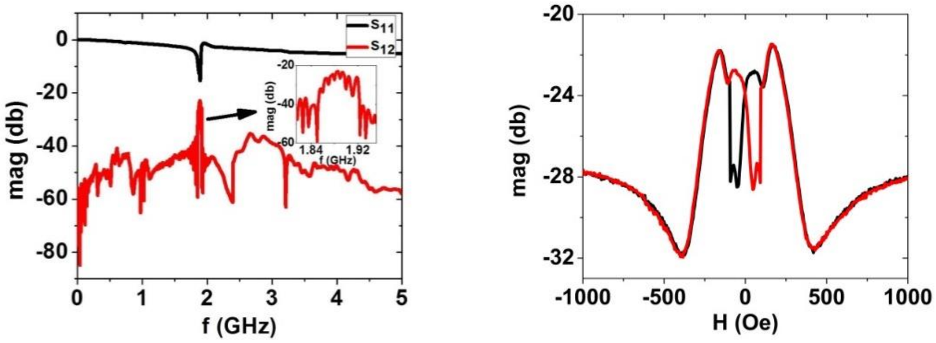

(c) (d)

Fig.S1 Acoustically driven ferromagnetic resonance experiment: a) The device schematic showing two IDTs and a big ferromagnetic pad in between. b) The SEM image of the IDT lines, it was a symmetric design with the width of the lines around 400 nm and the periodicity around 2 μm. c) The S_{11} and S_{12} scattering parameters of the device. S_{11} shows a dip and S_{12} shows a peak at around 1.9 GHz indicating transmission of SAW. The inset shows the zoomed in peak of S_{12} d) The variation of S_{12} as a function of external magnetic field which was applied at an angle of 45^0. The red and the black curves are for the dual sweep of the magnetic field. The hysteresis around zero field value is because of magnetization switching and the symmetric dips at ±450 Oe correspond to the FMR.

2) Generation of SAW by FMR

Here we analyze the excitation of magnetization by SAW and its inverse viz. generation of SAW by oscillating magnetization. The analysis is restricted to the present experimental case. In particular we will consider only in-plane external magnetic field. The reference frames used in the analysis are shown in the Fig.1 (and Fig.S1). The z (same as z') axis is out of plane. We assume in-plane magnetic field applied at an angle θ_H w.r.t x-axis. The resultant equilibrium magnetization is along angle θ w.r.t x axis. The x'-axis is taken along the direction of equilibrium magnetization. The free energy density of the FM is taken as: $(\mu_0 M_s)[-\hat{m}.\overline{H}_{ext} - (1/2)H_{//}{m_x}^2 + (1/2)H_\perp {m_z}^2]$. $\hat{m}$ denotes unit vector along magnetization direction and M_s denotes saturation magnetization of FM. Thus positive values of $H_{//}$ and $H_\perp$ correspond to x axis as easy axis, and z axis as out of plane hard axis. The magnetic field acting on the FM is given by: $\overline{H} = -(1/\mu_0 M_s)(\partial F/\partial \hat{m}) = \overline{H}_{ext} + H_{//} m_x \hat{x} - H_\perp m_z \hat{z}$. The magnetization dynamics is governed by LLG equation as: $\partial_t \hat{m} = -\gamma_0(\hat{m}\times\overline{H}) + \alpha(\hat{m}\times\partial_t\hat{m})$, where γ_0 denotes the gyromagnetic ratio and α denotes damping factor.

When ac voltage is applied to IDTs (port 1), it generates SAW along ±x direction. The wave travelling along x direction is incident on a Ni/Au line (port 2) as shown in the Fig.1. (For simplicity we will consider only one Ni/Au line at port 2.) The input wave is given by

$$\phi_{in}(x,t) = \phi_{in}\exp(ikx)\exp(-i\omega t) --- \quad (S1)$$

Where ϕ denotes the surface potential associated with wave. The corresponding surface displacement is related to the surface potential by $u_x = c_x\phi$, where c_x is a constant for given substrate. (We neglect here the fact that SAW in $LiNbO_3$ substrate have displacements also along z direction, and simply take a single component along x direction. Similar assumptions have been made in ref S2.) The strain associated with the wave is then given by, $\varepsilon \equiv \varepsilon_{xx} = \partial_x u_x$. The contribution of SAW to the free energy density of the FM is given by: $\mu_0 M_s b_1 \varepsilon(t) {m_x}^2$ [S1, S2]. This gives rise to an effective magnetic field along x direction given by:

$$h_x = -(1/\mu_0 M_s)(\partial F/\partial m_x) = -2b_1\varepsilon(t)m_{x0} --- (S2)$$

which excites magnetization of FM line. (The index 0 on m_x denotes the equilibrium value.) The y' component of magnetization is given as: $m_{y'} = \chi_{11}h_{y'}$, where $h_{y'}$ is related to h_x by $h_{y'} = -h_x\sin\theta$, and χ denotes the susceptibility. Further we can write, $\delta m_x = -\sin\theta\, m_{y'}$. i.e.

$$\delta m_x = \sin^2\theta\,\chi_{11}\,h_x --- (S3)$$

Equations S2 and S3 show that the incident SAW along x direction applies effective magnetic field along x-axis proportional to the equilibrium value of m_x, and also gives rise to oscillation of m_x.

We expect that conversely, oscillating m_x would excite SAW along x direction, with amplitude proportional to equilibrium value of m_x (and coupling constant b_1) i.e.

$$\varepsilon = C b_1 m_{x0} \delta m_x \; --- \quad (S4)$$

where C is a constant. We will find out the value of C from power consideration, and later show that above equation also gives the correct relation between S_{12} and S_{21}.

Calculation of C:

Consider a SAW incident on Ni line as in above section. The power carried by the input wave is given by [S3]:

$$P_{in} = \frac{|\phi_{in}|^2}{2z_0}\frac{W}{\lambda} = \frac{1}{k^2|c_x|^2}\frac{|\varepsilon_{in}|^2}{2z_0}\frac{W}{\lambda} \; --- \quad (S5)$$

where W is the beam width (same as length of FM), z_0 is the characteristic impedance of SAW substrate. We have used the relation, $\varepsilon = \partial_x u_x = c_x (ik)\phi$.

As discussed above, the SAW gives rise to magnetization oscillation. The average energy loss per unit time due to magnetization oscillation can be written as:

$$power\, loss = (1/2)\,\mu_0 M_s vol \;\; \omega\, \mathrm{Im}(\chi_{11}) \left|h_{y'}\right|^2$$
$$= 2\,\mu_0 M_s vol \;\; \omega\, \mathrm{Im}(\chi_{11}) b_1^2 \sin^2\theta \cos^2\theta \left|\varepsilon_{in}\right|^2 \; --- \quad (S6)$$

We can define a complex power loss by replacing $\mathrm{Im}(\chi_{11})$ in above equation by $-i\chi_{11}$ [S1]. This power loss changes the amplitude (and phase) of the transmitted SAW. We can think of this in the following way: The oscillating magnetization emits a SAW and the transmitted SAW is given by superposition of incident wave and emitted wave. We now assume that at resonance, there is no phase change of the transmitted wave. If the wave emitted by FM has amplitude of ε_1 (phase same as incident wave), we can write

$$transmitted\;\; power = \frac{|\varepsilon_{in} + \varepsilon_1|^2}{2z_0}\frac{1}{k^2|c_x|^2}\frac{W}{\lambda} \approx \{|\varepsilon_{in}|^2 + 2\varepsilon_1\varepsilon_{in}\}\,\frac{1}{2z_0}\frac{1}{k^2|c_x|^2}\frac{W}{\lambda}$$
$$\Rightarrow power\;\; loss = 2\varepsilon_1\varepsilon_{in}\frac{1}{2z_0}\frac{1}{k^2|c_x|^2}\frac{W}{\lambda} \; --- \quad (S7)$$

Equating the power loss expressions S6 and S7, we get

$$\varepsilon_1 = -z_0 \frac{k^2|c_x|^2\lambda}{W}\mu_0 M_s vol \;\; \omega\, \mathrm{Im}(\chi_{11}) \;\; (2b_1^2 \;\sin^2\theta\cos\theta^2)\,\varepsilon_{in} \; --- \quad (S8.1)$$

In general using the complex power loss, we can write

$$\varepsilon_1 = -z_0 \frac{k^2|c_x|^2\lambda}{W}\mu_0 M_s vol \;\; \omega\,(-i\chi_{11}) \;\; (2b_1^2 \;\sin^2\theta\cos\theta^2)\,\varepsilon_{in} \; --- \quad (S8.2)$$

The above equation implies that in general the wave emitted by FM has a phase shift w.r.t input wave. At resonance, $-i\chi_{11}$ is real positive number, implying that the emitted wave is out of phase by 180°.

We have from equation (S4)

$$\varepsilon_1 = C\, b_1\, m_{x0}\, \delta m_x = -2C\, b_1^{\,2} \cos^2\theta \sin^2\theta\, \chi_{11}\ \ \varepsilon_{in}$$
$$using\ \ m_{x0} = \cos\theta \ \ and \ \ \delta m_x = -2b_1\, \varepsilon_{in} \cos\theta \sin^2\theta\, \chi_{11}$$

Comparing with equation (S8.2), we get the value of C as:

$$C = -i\, z_0 \frac{k^2 |c_x|^2 \lambda}{W}\, \omega \mu_0 M_s vol \ --- \quad (S9.1)$$

From equations S4 and S9.1, we can write:

$$\varepsilon_1 = Ab_1\ m_{x0} \frac{dm_x}{dt} \ where\ A = z_0 \frac{k^2 |c_x|^2 \lambda}{W}\, \mu_0 M_s vol --- \quad (S9.2)$$

It should be noted that the oscillating magnetization emits wave in both ±x direction with the same amplitude ε_1. One way to see this, is as follows: The effective magnetic field arising from the input SAW is determined by ε(t) at the position of FM. It does not depend on whether the input wave came from +x or –x direction. The emission of surface acoustic waves by oscillating magnetization is shown schematically in Fig.S2.

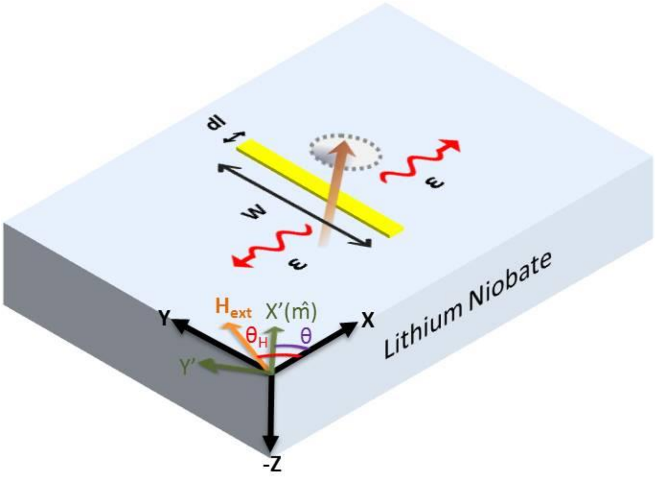

Fig.S2 SAW generation by oscillating magnetization. The magnetization generates SAW in both the directions with equal amplitudes.

The expression derived for emission of SAW by oscillating magnetization is valid for a single thin FM line (*dl*<<λ, see Fig.S2). The effective field from input SAW on the FM is uniform in this case, and we assumed a macro-spin precession. In the case many thin uncoupled lines (i.e. neglecting dipolar and exchange coupling between them), we should take superposition of waves emitted by each thin line.

3) Calculation of S parameters:

The two ports are coupled via the FM line. (We again consider a single thin line instead of 100 lines.) We now consider the receiving and transmitting characteristics of the IDT port. A circuit representation of the IDT port is shown below [S3].

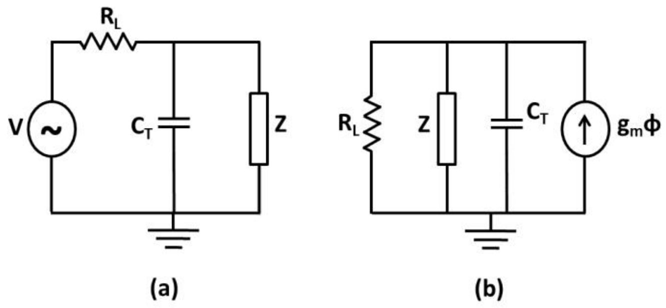

Fig.S3 Circuit representation of the IDT port as transmitter and receiver [S3]

C_T denotes the capacitance of the IDTs, and Z represents a combination of acoustic impedance and magnetic impedance. R_L denotes the resistance of network analyzer (50 Ω). V denotes the input voltage from network analyzer, and ϕ denotes the surface potential associated with SAW. Consider a case where port 1 is excited by network analyzer. The voltage V_T across the capacitor is given by $V_T = 2Z_1 V_{in}/(R_L + Z_1)$, where V_{in} the voltage applied by network analyzer and Z_1 denotes combined impedance of C_T and Z. The IDTs generate SAW with amplitude, $\phi = \mu V_T$, where μ denotes the transmitter response function of the IDTs. The amplitude of the strain of the SAW travelling towards ports 2 (i.e. x direction) can be written as:

$$\varepsilon = \mu(ikc_x)\frac{2Z_1}{R_L + Z_1}V_{in} --- (S10)$$

This wave excites the magnetization of FM line as discussed above. The oscillation of x-component of magnetization is given by:

$$\delta m_x = -2b_1 \cos\theta \sin^2\theta\, \chi_{11}\, \varepsilon = -2b_1 \cos\theta \sin^2\theta\, \chi_{11}\, \mu(ikc_x)\frac{2Z_1}{R_L + Z_1}\exp(ikl)V_{in} --- \quad (S11)$$

where *l* denotes the distance between IDT centre and FM line. The phase factor of *exp(ikl)* accounts for the fact that SAW generated at x=0 needs to travel distance *l* to interact with FM line. The above equation relates the input voltage from network analyzer to the excitation of x component of magnetization.

We now consider the receiving characteristics of IDTs. If a SAW with amplitude ϕ is incident on the IDTs from the port 2 side, the voltage measured by network analyzer is given by (see Fig.S3(b) above):

$$V_{out} = g_m \phi_{in} \frac{R_L Z_1}{R_L + Z_1}$$

where g_m denotes the receiver response function of the IDTs. If the magnetization of FM is oscillating and it emits SAW along x direction, given by $\varepsilon = C b_1 m_{x0}\, \delta m_x$ (See equation (S4)) This corresponds to a surface potential of $\phi = \varepsilon/(ikc_x^*)$. Thus the voltage measured by the network analyzer is given by:

$$V_{out} = \frac{g_m}{ikc_x^*}\frac{R_L Z_1}{R_L + Z_1}\varepsilon \exp(ikl) = \frac{g_m}{ikc_x^*}\frac{R_L Z_1}{R_L + Z_1} C\, b_1\, m_{x0}\, \delta m_x \exp(ikl) --- \quad (S12)$$

The phase factor of *exp(ikl)* again accounts for the fact that SAW generated at FM line needs to travel distance *-l* to reach the IDT centre. The above equation relates the magnetization oscillation (δm_x) to the voltage measured at port 1.

We now consider transmitting characteristics of port 2. When port 2 is excited by voltage (V_{in}) from network analyzer, the current flowing in the Au lines is given by, $I = 2V_{in}/(R_L + Z_2)$, where Z_2 includes the wire resistance, magnetic and acoustic impedances. This current produces a magnetic field along x direction ($h_x = I/2dl$) and excites the magnetization as:

$$hy' = -\sin\theta\, h_x,\ m_{y'} = \chi_{11} h_{y'},\ \ \delta m_x = -\sin\theta\, m_{y'} = \sin^2\theta\, \chi_{11}\, h_x$$

$$\Rightarrow \delta m_x = \sin^2\theta\, \chi_{11} \frac{V_{in}}{dl(R_L + Z_2)} --- \quad (S13)$$

Now consider the receiving characteristics of port 2. The flux in the port 2 circuit from the magnetization of FM line is [S4], $\varphi = (1/2)\mu_0 M_s W t\, \delta m_x$, where t is the thickness of FM. If the magnetization is oscillating, it induces an emf in the circuit. The voltage measured by network analyzer is given by,

$$V_{out} = \frac{-\partial\varphi}{\partial t}\frac{R_L}{R_L + Z_2} = i\omega \frac{1}{2}\mu_0 M_s W t \frac{R_L}{R_L + Z_2}\delta m_x --- \quad (S14)$$

We can now calculate S_{21} and S_{12} as follows. When voltage is applied to port 1 by network analyzer, it excites the magnetization of FM line (equation (S11)), which in turn induces a voltage in port 2 (equation (S14)). Thus S_{21}, which is the ratio of induced voltage in port 2 to voltage applied to port 1, is given by

$$\begin{aligned} S_{21} &= \{i\omega \frac{1}{2}\mu_0 M_s W t \frac{R_L}{R_L + Z_2}\}\{-2b_1 \cos\theta \sin^2\theta\, \chi_{11}\, \mu (ikc_x) \frac{2Z_1}{R_L + Z_1}\exp(ikl)\} \\ &= b_1 (\mu c_x) k\, \omega\, \chi_{11} (\mu_0 M_s W t) \frac{2R_L Z_1}{(R_L + Z_2)(R_L + Z_1)} \exp(ikl) \cos\theta \sin^2\theta --- \quad (S15) \end{aligned}$$

Similarly, S_{12} which is the ratio of induced voltage in port 1 to voltage applied to port 2, is given from equation (S12) and equation (S13), as

$$\begin{aligned} S_{12} &= \{\frac{g_m}{ikc_x^*}\frac{R_L Z_1}{R_L + Z_1} A b_1\, m_{0x} \exp(ikl)\}\{\sin^2\theta\, \chi_{11} \frac{1}{dl(R_L + Z_2)}\} \\ &= -b_1 (\mu c_x) k\, \omega\, \chi_{11} (\mu_0 M_s W t) \frac{2R_L Z_1}{(R_L + Z_2)(R_L + Z_1)} \exp(ikl) \cos\theta \sin^2\theta --- \quad (S16) \end{aligned}$$

We have used value of C from equation (S9.1), and put m_{x0}=cosθ. We have also used the relation between the transmitter and receiver response functions of IDTs as, $g_m = (2\mu / z_0)(W/\lambda)$ [S3].

Thus we get the relation $S_{12}(\theta) = -S_{21}(\theta)$. Further the angular dependence of S_{12} is $\cos\theta \sin^2\theta$, which agrees with experimental observation. Further, we can see that $S_{12}(\theta) = -S_{12}(180+\theta)$. Thus $S_{12}(\theta) = S_{21}(180+\theta)$ i.e. $S_{12}(B) = S_{21}(-B)$.

4) Numerical estimation of S_{21} signal

We could use the equation S15 to evaluate S_{21} signal. But we need to know the capacitance of the IDTs. Instead, we will follow a different approach. We estimate the amplitude of SAW generated using $|S_{11}|$ data [S5]. We see a dip in $|S_{11}|$ at 1.89 GHz as shown in Fig. S1c. We take the change in $|S_{11}|^2$ as the power loss due to the emission of SAW in both ±x directions. The power carried by the SAW travelling in +x direction is, $0.5P \times \Delta|S_{11}|^2$. We can then use the equation S5 to calculate the strain ε. Using P=5 dBm, $\Delta|S_{11}|^2$=0.5, z_0=4.76 kΩ [S3], c_x=-1.8 i A°/V [S3], we get ε=2.29X10^{-4}. Using b_1=25 T [S2] and $\theta_H = 45^o$, we get the effective rf magnetic field created by SAW along x axis to be 1.19 Oe. The voltage induced in the port 2 can be calculated using equations (S3) and (S14), with parameters: ω=2π*1.89X10^9, Ms=800 emu/cc, α=0.05, W=90 µm, t=20 nm, R_L=50 Ω, Z_2=30 Ω and H_{ext}=120 Oe (which corresponds to resonance). We can then get S_{12} as ratio of induced voltage to applied voltage comes out to be, $|S_{12}|$=1.2X10^{-4}, which is comparable to the observed value (see Fig. S4).

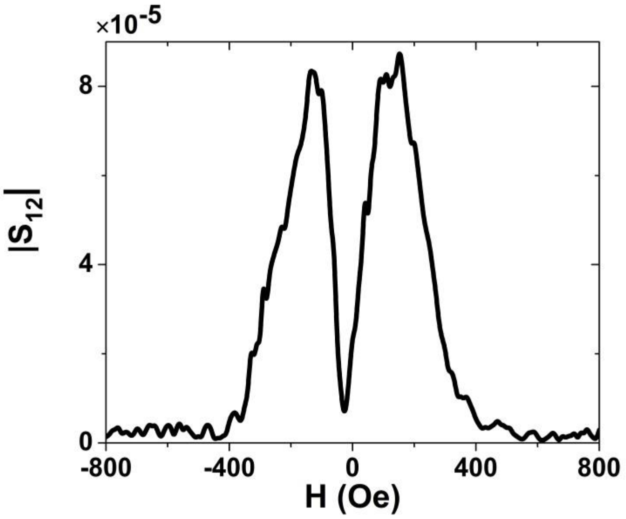

Fig.S4 Variation of S_{12} magnitude with field for a fixed frequency of the IDT (1.9 GHz)

5) About S_{12} and S_{21} spectrum shape

It's a bit surprising that the real and imaginary parts of S_{12} (or S_{21}) both look like peaks (see Fig.3) or dips. This happens due to the phase factor *exp(ikl)* in equation (S15) and (S16). Fig.S5 shows S_{12} data of Fig.3, multiplied by exp(iΦ), where we chose Φ=65°. Now we can see the expected peak-dispersion form. It can be seen from equation (S15) or (S16) that apart from χ_{11}, the factor $\sin^2(\theta)\cos(\theta)$ also depends on applied field, which distorts the signal shape.

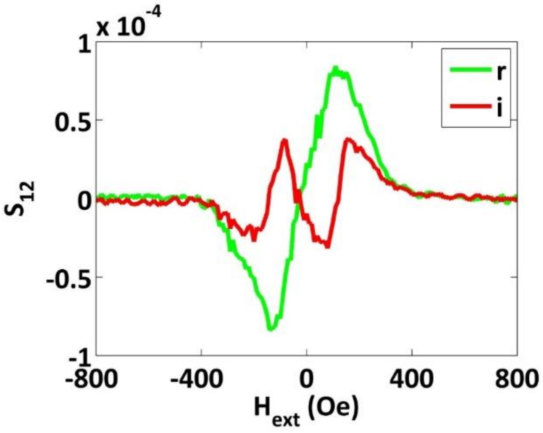

Fig.S5 Real and imaginary parts of S_{12} for a different phase value, the green curve marked as ‘r’ and the red curve marked as ‘i’ show the real and imaginary parts respectively.

6) **S-parameters of the device for 3 GHz frequency and for device with s=λ/2**

The S_{12} signal for 3GHz frequency 3 GHz is shown in Fig.S6(a). The FMR field for 3 GHz is ±315 Oe (Fig.2). We don’t see any clear peak or dip in S_{12} around this field value. This is because even if we have FMR and generation of SAW at 3 GHz from each Ni line, the corresponding wavelength (λ=1.33μm) does not match the periodicity of transmitting and receiving ports.

We also measured S-parameters of the device with s=λ/2 using 1.89 GHz frequency and θ_H=45°.The results are shown in Fig.S6(b). Even if we have FMR and generation of surface acoustic waves from each Ni line, because of the periodicity, there is destructive interference and no wave propagates towards the IDTs.

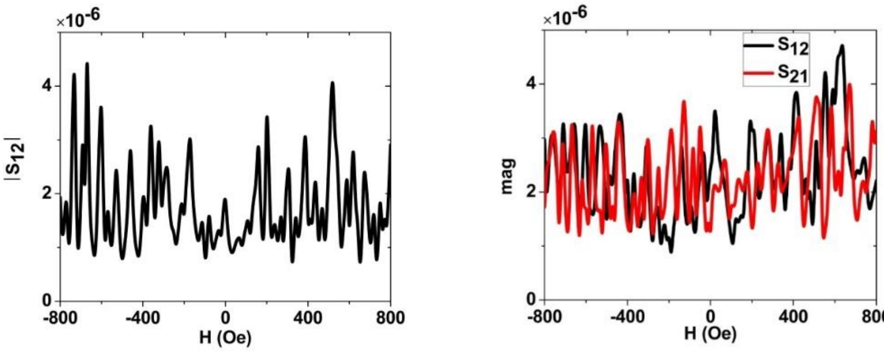

(a) (b)

*Fig.S6 (a) Magnitude of S_{12} signal for 3 GHz frequency (s=*λ device) *(b) S_{12} and S_{21} signals for s=*λ/2 device at 1.89 GHz frequency, the field was applied at 45^0 for all these measurements.

References:

[S1] Dreher, L. et al. Surface acoustic wave driven ferromagnetic resonance in nickel thin films: Theory and experiment. Phys. Rev. B 86, 134415 (2012)

[S2] Weiler, M. et al. Elastically driven ferromagnetic resonance in nickel thin films. Phys. Rev. Lett. 106, 117601 (2011)

[S3] Datta, S. *Surface Acoustic Wave Devices*. Prentice Hall, Englewood Cliffs, Michigan (1986)

[S4] Silva, T. J., Lee, C.S., Crawford, T. M. & Rogers, C. T. Inductive measurement of ultrafast magnetization dynamics in thin-film Permalloy. J. Appl. Phys. 85, 11 (1999)

[S5] Weiler, M. et al., Spin Pumping with Coherent Elastic Waves, Phys. Rev. Lett. 108, 176601 (2012)
